# Crystal structure of a π-conjugated N-donor ligand-bridged anionic bis­muth(III) iodide one-dimensional coordination polymer

**DOI:** 10.1107/S2056989026001362

**Published:** 2026-02-27

**Authors:** Naohiro Takahashi, Yuki Endo, Hiyori Sasaki, Takashi Okubo

**Affiliations:** a Department of Chemistry, Kindai University, Osaka, Japan; b Department of Energy and Materials, Kindai University, Osaka, Japan; Tokyo University of Science, Japan

**Keywords:** crystal structure, bis­muth iodide, coordination polymer

## Abstract

The crystal structure of a one-dimensional anionic bis­muth(III) iodide coordination polymer, *catena*-poly[[tri­iodido­bis­muth(III)]-di-μ-iodido-[tri­iodido­bis­muth(III)]-μ-4,7-bis­(pyridin-4-yl)benzo[*c*][1,2,5]thia­diazole], {(C_16_H_36_N)_2_[Bi_2_I_8_(C_16_H_10_N_4_S)]}_*n*_, constructed from μ_2_-iodido-bridged dinuclear [Bi_2_I_8_]^2−^ units and the π-conjugated N-donor ligand 4,7-bis­(pyridin-4-yl)benzo[*c*][1,2,5]thia­diazole (dpbt) is reported. Each Bi^III^ centre adopts a distorted octa­hedral geometry with five iodido ligands and one nitro­gen donor atom. The dinuclear units are linked by dpbt to form infinite chains, with charge balance provided by tetra-*n*-butyl­ammonium cations. The crystal packing is governed by C—H⋯I hydrogen bonds and C—H⋯π inter­actions, while π–π stacking is suppressed by steric effects. Anionic iodido­bis­muth polymers of this type are rare.

## Abstract 2

A π-conjugated N-donor ligand links μ_2_-iodido-bridged dinuclear [Bi_2_I_8_]^2−^ units to afford a rare one-dimensional anionic iodido­bis­muth(III) coordination polymer, which was synthesized and structurally characterized by single-crystal X-ray diffraction.

## 1. Chemical context

Bismuth iodide compounds have attracted considerable attention in catalysis, bioinorganic chemistry and materials science owing to their structural diversity and unique electronic properties (Fu *et al.*, 2021 ▸; Hrizi *et al.*, 2025 ▸; Ran *et al.*, 2017 ▸). Bismuth(III) triiodide complexes are known to exhibit a wide variety of coordination modes, ranging from mononuclear species (Travis *et al.*, 2016 ▸; Deuter *et al.*, 2025 ▸) to dinuclear clusters (Tershansy *et al.*, 2006 ▸; Bhatia *et al.*, 2024 ▸) and extended coordination polymers formed through iodido bridging (Ozturk *et al.*, 2019 ▸; Kelly *et al.*; 2018 ▸). However, examples of anionic iodido­bis­muth-based coordination polymers remain relatively limited. Such systems are attractive because the large and highly polarizable iodide ligands promote flexible coordination modes and diverse structural architectures. In addition, bis­muth halides have attracted considerable inter­est owing to their optical and semiconducting properties, as well as their potential as lead-free functional materials. Therefore, the exploration of new iodido­bis­muth coordination polymers is important from both structural and application-oriented perspectives. Neutral N-donor bridging ligands such as pyridyl-based linkers are effective structure-directing components for constructing low-dimensional bis­muth–halide frameworks. In this context, we investigated the reaction of BiI_3_ with iodide ions and the bridging ligand 4,7-bis­(pyridin-4-yl)benzo[*c*][1,2,5]thia­diazole (dpbt). This approach afforded an anionic iodido­bis­muth(III) coordination polymer, and herein we report its crystal structure.
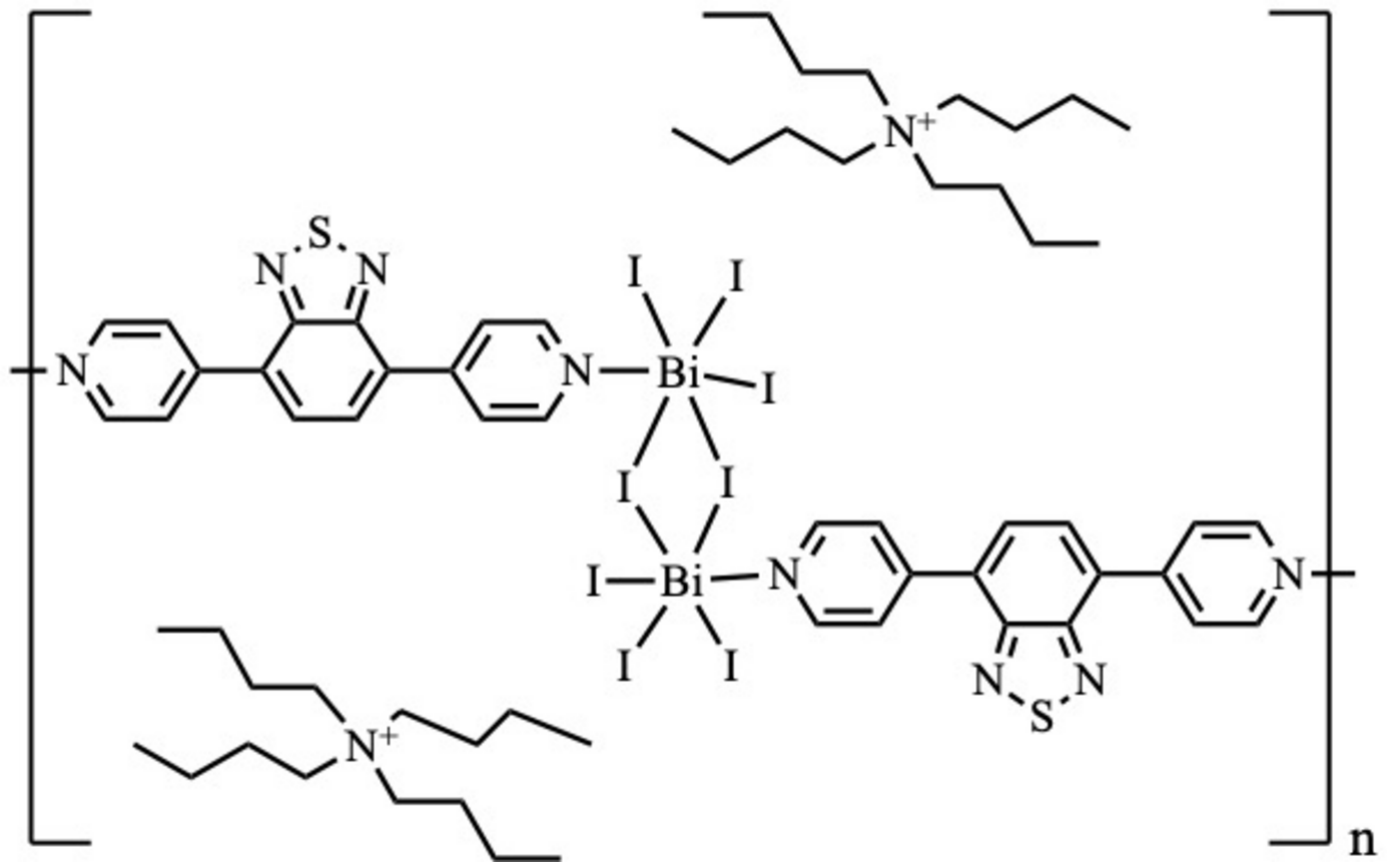


## 2. Structural commentary

**Figure 1:**
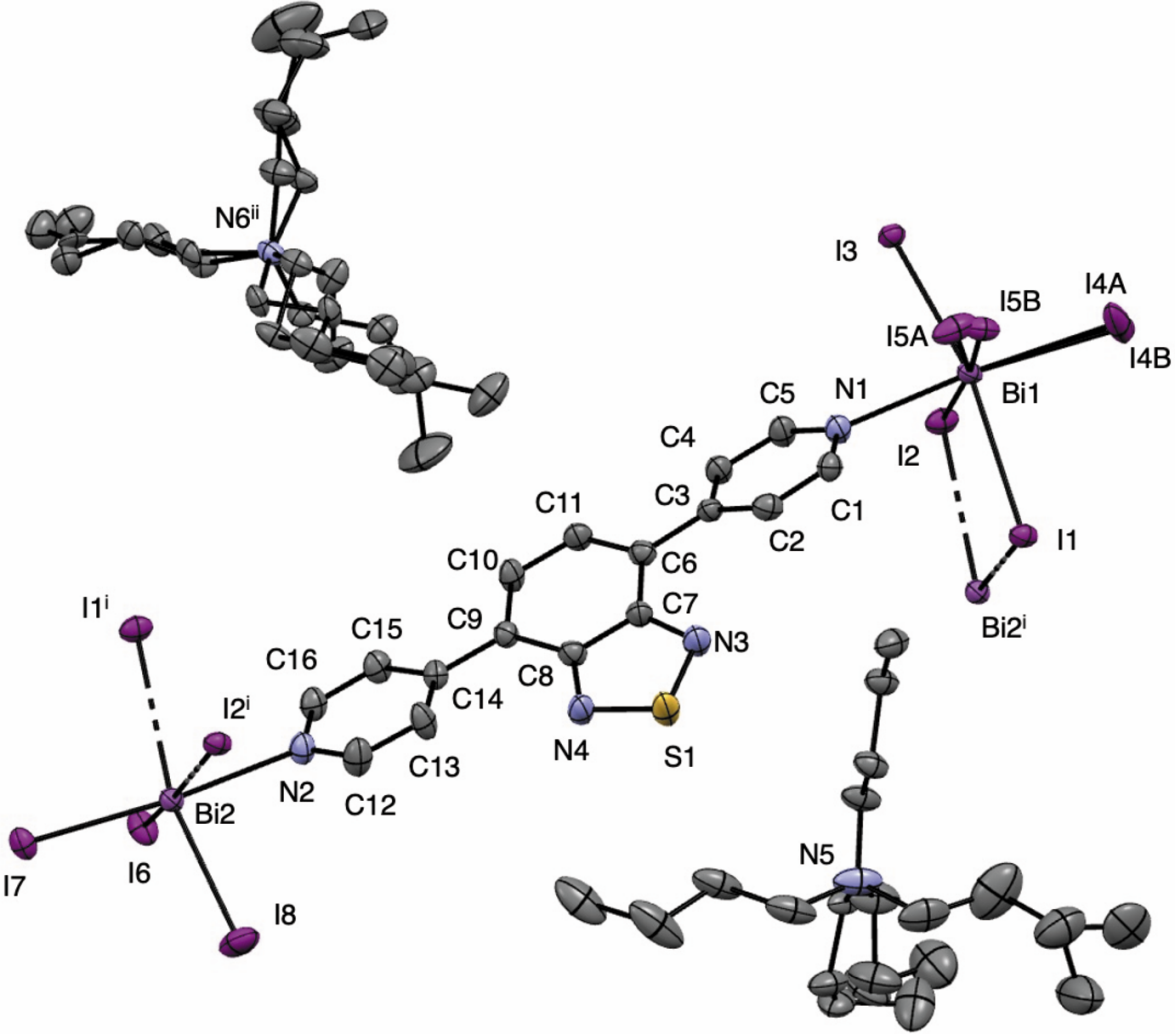
View of anionic one-dimensional Bi^III^ coordination polymer with 50% probability level ellipsoids: Bi, light purple; I, purple; S, yellow; C, gray; and N, blue. Hydrogen atoms are omitted for clarity. Symmetry codes: (i) *x*, *y* − 1, *z*; (ii) −*x* + 1, *y* − 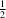, −*z* + 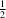.

Single-crystal X-ray diffraction analysis reveals that the title compound consists of μ_2_-iodido-bridged dinuclear [Bi_2_I_8_]^2−^ building units linked by dpbt ligands to form a one-dimensional anionic coordination polymer (Fig. 1 ▸). Each Bi^III^ centre adopts a distorted octa­hedral coordination environment defined by five iodido ligands and one nitro­gen donor atom from a dpbt ligand. The Bi—I bond distances fall in the range 2.859 (4)–3.2756 (4) Å, while the Bi—N bond lengths are 2.655 (5) and 2.671 (6) Å (Table 1 ▸). The bond angles around the Bi centres range from 83.90 (1) to 100.4 (3)°, deviating from the ideal 90° angles expected for a regular octa­hedron. The observed distortion is attributed to the asymmetric μ_2_-iodido bridging and the stereochemically active 6*s*^2^ lone pair on the Bi^III^ centre, which is known to induce angular compression in bis­muth halide complexes. The bridging Bi—I bonds are slightly longer than the terminal Bi—I bonds, in agreement with previously reported iodido­bis­muth structures (Ramler *et al.*, 2022 ▸). Two Bi^III^ centres are connected through μ_2_-iodido bridges to form a dinuclear unit, with an intra­dimer Bi⋯Bi separation of 4.6894 (7) Å. This distance exceeds the sum of the van der Waals radii of bis­muth (4.0 Å), indicating the absence of significant Bi⋯Bi metallophilic inter­actions. The dpbt ligand acts as a linear N,N-bridging linker connecting adjacent dinuclear units, thereby generating a one-dimensional polymeric chain. The ligand is slightly non-planar, with dihedral angles of 16.80 (1) and 22.83 (1)° between the pyridyl and benzo­thia­diazole ring planes. The negative charge of the inorganic chain is compensated by tetra-*n*-butyl­ammonium (TBA^+^) counter-cations located in the crystal.

## 3. Supra­molecular features

**Figure 2:**
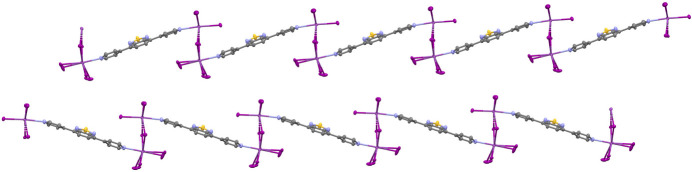
A view of the one-dimensional coordination polymer formed by μ_2_-iodido-bridged dinuclear iodido­bis­muth(III) units linked by dpbt ligands. Tetra-*n*-butyl­ammonium cations and hydrogen atoms are omitted for clarity.

In the crystal, the one-dimensional chains are arranged parallel (Fig. 2 ▸) to each other and are consolidated by weak inter­molecular inter­actions. C—H⋯I hydrogen bonds (Table 2 ▸) are observed between the iodido ligands and the alkyl chains of the tetra-*n*-butyl­ammonium (TBA^+^) cations. In addition, C—H⋯π inter­actions between the benzo­thia­diazole moieties of the bridging ligands and the alkyl chains of the TBA^+^ cations contribute to the three-dimensional packing of the structure. Owing to the steric bulk of the TBA^+^ cations, no significant π–π stacking inter­actions between the ligands are observed.

## 4. Database survey

A search of the Cambridge Structural Database (CSD, version 6.01, update of November 2025; Groom *et al.*, 2016 ▸) revealed several iodido­bis­muth(III) coordination polymers constructed through μ_2_-iodido bridges and N-donor ligands. Most of the reported examples are neutral (Sorg *et al.*, 2018 ▸; Ozturk *et al.*, 2019 ▸). A few anionic coordination polymers based on mononuclear iodido­bis­muth units linked by bridging ligands have also been reported (Kelly *et al.*, 2017 ▸), but such examples remain rare. The present compound represents a rare example of an anionic iodido­bis­muth(III) coordination polymer assembled from μ_2_-iodido-bridged dinuclear units with N-donor ligands.

## 5. Synthesis and crystallization

A mixture of BiI_3_ (59 mg, 0.1 mmol) and tetra­butyl­ammonium iodide (37 mg, 0.1 mmol) was dissolved in acetone (15 mL), and dpbt (29 mg, 0.1 mmol) was added to the solution under stirring. The resulting solution was combined with di­ethyl ­ether (Et_2_O) in a 1:1 (*v*/*v*) ratio, transferred into an 8 mm diameter glass tube, sealed, and allowed to stand at room temperature for several days. Orange block-shaped crystals suitable for single-crystal X-ray analysis were obtained.

## 6. Refinement

Crystal data, data collection and structure refinement details are summarized in Table 3 ▸. Residual electron density (2.54 e Å^−3^) was found 0.83 Å from atom I7, suggesting slight positional disorder of the iodido ligand. An attempt was made to model this atom over two positions; however, the disorder model did not improve the refinement statistics and resulted in an unstable minor occupancy. Therefore, no disorder model was applied and the atom was refined as an ordered site. The residual density remained within acceptable limits after refinement.

## Supplementary Material

Crystal structure: contains datablock(s) global, I. DOI: 10.1107/S2056989026001362/jp2025sup1.cif

Structure factors: contains datablock(s) I. DOI: 10.1107/S2056989026001362/jp2025Isup2.hkl

CCDC reference: 2529756

Additional supporting information:  crystallographic information; 3D view; checkCIF report

## Figures and Tables

**Table 1:** Selected geometric parameters (Å, °)

Bi2—I2^i^	3.2756 (4)	Bi1—I3	2.9840 (4)
Bi2—I7	2.9571 (5)	Bi1—I1	3.1292 (5)
Bi2—I1^i^	3.2267 (4)	Bi1—I5	2.9657 (19)
Bi2—I6	2.9290 (5)	Bi1—I4	3.0143 (13)
Bi2—I8	2.9489 (5)	Bi1—N1	2.655 (5)
Bi2—N2	2.671 (6)	Bi1—I4*A*	2.859 (4)
Bi1—I2	3.1757 (4)	Bi1—I5*A*	3.131 (5)
			
I7—Bi2—I2^i^	92.778 (12)	I5—Bi1—I2	93.81 (5)
I7—Bi2—I1^i^	97.092 (13)	I5—Bi1—I3	93.08 (6)
I1^i^—Bi2—I2^i^	83.901 (11)	I5—Bi1—I4	96.54 (3)
I6—Bi2—I7	94.290 (13)	I4—Bi1—I2	91.10 (4)
I6—Bi2—I1^i^	88.675 (13)	I4—Bi1—I1	88.49 (4)
I6—Bi2—I8	97.771 (17)	N1—Bi1—I2	83.58 (12)
I8—Bi2—I2^i^	88.211 (15)	N1—Bi1—I3	86.32 (12)
I8—Bi2—I7	94.710 (16)	N1—Bi1—I1	84.81 (13)
N2—Bi2—I2^i^	84.97 (13)	N1—Bi1—I5	90.27 (14)
N2—Bi2—I6	87.45 (13)	N1—Bi1—I5*A*	81.4 (2)
N2—Bi2—I8	89.75 (12)	I4*A*—Bi1—I2	91.29 (10)
I3—Bi1—I1	85.099 (12)	I4*A*—Bi1—I3	99.40 (8)
I3—Bi1—I4	98.12 (4)	I4*A*—Bi1—I1	100.4 (3)
I3—Bi1—I5*A*	89.26 (7)	I4*A*—Bi1—I5*A*	93.80 (15)
I1—Bi1—I2	87.168 (12)	I5*A*—Bi1—I2	95.97 (7)

Symmetry code: (i) 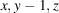.

**Table 2:** Hydrogen-bond geometry (Å, °) *Cg* is the centroid of the benzo­thia­diazole ring.

*D*—H⋯*A*	*D*—H	H⋯*A*	*D*⋯*A*	*D*—H⋯*A*
C39^ii^—H39*D*^ii^⋯I3	0.97	2.98	3.76 (4)	157
C37^ii^—H37*A*^ii^⋯I3	0.99	3.03	3.966 (8)	138
C34^ii^—H34*D*^ii^⋯I4	0.99	2.89	3.71 (3)	141
C36^ii^—H36*E*^ii^⋯I4	0.98	2.48	3.41 (5)	158
C37^i^—H37*C*^i^⋯I7	0.99	3.03	3.96 (2)	157
C40—H40*A*⋯*Cg*	0.98	2.86	3.56 (2)	129

Symmetry codes: (i) 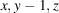; (ii) 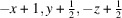.

**Table 3:** Experimental details

Crystal data	
Chemical formula	(C_16_H_36_N)_2_[Bi_2_I_8_(C_16_H_10_N_4_S)]
*M*_r_	2208.41
Crystal system, space group	Monoclinic, *P*2_1_/*c*
Temperature (K)	86
*a*, *b*, *c* (Å)	16.8421 (3), 16.1181 (2), 24.9419 (4)
β (°)	99.273 (1)
*V* (Å^3^)	6682.31 (18)
*Z*	4
Radiation type	Cu *K*α
μ (mm^−1^)	39.78
Crystal size (mm)	0.17 × 0.08 × 0.03

Data collection	
Diffractometer	XtaLAB Synergy R, HyPix
Absorption correction	Gaussian (*CrysAlis PRO*; Rigaku OD, 2025 ▸)
*T*_min_, *T*_max_	0.142, 0.640
No. of measured, independent and observed [*I* > 2σ(*I*)] reflections	48823, 13582, 12533
*R*_int_	0.041
(sin θ/λ)_max_ (Å^−1^)	0.632

Refinement	
*R*[*F*^2^ > 2σ(*F*^2^)], *wR*(*F*^2^), *S*	0.038, 0.099, 1.04
No. of reflections	13582
No. of parameters	811
No. of restraints	1016
H-atom treatment	H-atom parameters constrained
Δρ_max_, Δρ_min_ (e Å^−3^)	2.35, −1.40

Computer programs: *CrysAlis PRO* (Rigaku OD, 2025 ▸), *SHELXT* (Sheldrick, 2015*a* ▸), *SHELXL* (Sheldrick, 2015*b* ▸) and *OLEX2* (Dolomanov *et al.*, 2009 ▸).

## supplementary crystallographic information

### catena-Poly[[triiodidobismuth(III)]-di-µ-iodido-[triiodidobismuth(III)]-µ-4,7-bis(pyridin-4-yl)benzo[c][1,2,5]thiadiazole] Crystal data

**Table:**

(C_16_H_36_N)_2_[Bi_2_I_8_(C_16_H_10_N_4_S)]	*F*(000) = 4072
*M**_r_* = 2208.41	*D*_x_ = 2.195 Mg m^−^^3^
Monoclinic, *P*2_1_/*c*	Cu *K*α radiation, λ = 1.54184 Å
*a* = 16.8421 (3) Å	Cell parameters from 32276 reflections
*b* = 16.1181 (2) Å	θ = 2.6–77.2°
*c* = 24.9419 (4) Å	µ = 39.78 mm^−^^1^
β = 99.273 (1)°	*T* = 86 K
*V* = 6682.31 (18) Å^3^	Block, orange
*Z* = 4	0.17 × 0.08 × 0.03 mm

### catena-Poly[[triiodidobismuth(III)]-di-µ-iodido-[triiodidobismuth(III)]-µ-4,7-bis(pyridin-4-yl)benzo[c][1,2,5]thiadiazole] Data collection

**Table:**

XtaLAB Synergy R, HyPix diffractometer	13582 independent reflections
Radiation source: Rotating-anode X-ray tube, PhotonJet R (Cu) X-ray Source	12533 reflections with *I* > 2σ(*I*)
Mirror monochromator	*R*_int_ = 0.041
Detector resolution: 10.0000 pixels mm^-1^	θ_max_ = 76.9°, θ_min_ = 2.7°
ω scans	*h* = −19→21
Absorption correction: gaussian (CrysAlisPro; Rigaku OD, 2025)	*k* = −20→9
*T*_min_ = 0.142, *T*_max_ = 0.640	*l* = −31→31
48823 measured reflections	

### catena-Poly[[triiodidobismuth(III)]-di-µ-iodido-[triiodidobismuth(III)]-µ-4,7-bis(pyridin-4-yl)benzo[c][1,2,5]thiadiazole] Refinement

**Table:**

Refinement on *F*^2^	Primary atom site location: dual
Least-squares matrix: full	Hydrogen site location: inferred from neighbouring sites
*R*[*F*^2^ > 2σ(*F*^2^)] = 0.038	H-atom parameters constrained
*wR*(*F*^2^) = 0.099	*w* = 1/[σ^2^(*F*_o_^2^) + (0.0481*P*)^2^ + 49.057*P*] where *P* = (*F*_o_^2^ + 2*F*_c_^2^)/3
*S* = 1.04	(Δ/σ)_max_ = 0.002
13582 reflections	Δρ_max_ = 2.35 e Å^−^^3^
811 parameters	Δρ_min_ = −1.40 e Å^−^^3^
1016 restraints	

### catena-Poly[[triiodidobismuth(III)]-di-µ-iodido-[triiodidobismuth(III)]-µ-4,7-bis(pyridin-4-yl)benzo[c][1,2,5]thiadiazole] Special details

**Table:**

Geometry. All esds (except the esd in the dihedral angle between two l.s. planes) are estimated using the full covariance matrix. The cell esds are taken into account individually in the estimation of esds in distances, angles and torsion angles; correlations between esds in cell parameters are only used when they are defined by crystal symmetry. An approximate (isotropic) treatment of cell esds is used for estimating esds involving l.s. planes.

### catena-Poly[[triiodidobismuth(III)]-di-µ-iodido-[triiodidobismuth(III)]-µ-4,7-bis(pyridin-4-yl)benzo[c][1,2,5]thiadiazole] Fractional atomic coordinates and isotropic or equivalent isotropic displacement parameters (Å^2^)

**Table:**

	*x*	*y*	*z*	*U*_iso_*/*U*_eq_	Occ. (<1)
Bi2	0.27087 (2)	0.00184 (2)	0.34663 (2)	0.02186 (7)	
Bi1	0.30557 (2)	1.00171 (2)	0.16382 (2)	0.02376 (7)	
I2	0.15651 (2)	1.00224 (2)	0.22724 (2)	0.02430 (9)	
I3	0.45922 (2)	0.97354 (2)	0.12140 (2)	0.02451 (9)	
I7	0.27248 (2)	−0.18138 (3)	0.35281 (2)	0.02906 (10)	
I1	0.41537 (2)	1.02123 (3)	0.27689 (2)	0.02923 (10)	
I6	0.38856 (3)	0.02331 (3)	0.44573 (2)	0.03282 (10)	
I8	0.12403 (3)	0.02062 (4)	0.39589 (2)	0.04805 (13)	
I5	0.20537 (13)	0.96716 (18)	0.05710 (8)	0.0293 (4)	0.665 (11)
I4	0.30037 (16)	1.18817 (8)	0.15468 (6)	0.0306 (4)	0.665 (11)
S1	0.07341 (10)	0.52136 (12)	0.24614 (10)	0.0461 (5)	
N4	0.1312 (3)	0.4433 (4)	0.2658 (3)	0.0359 (14)	
N6	0.4915 (3)	0.7221 (3)	0.4789 (2)	0.0254 (10)	
N1	0.3097 (3)	0.8409 (3)	0.1872 (2)	0.0291 (12)	
N5	−0.0038 (4)	0.7295 (5)	0.4009 (3)	0.0472 (15)	
C3	0.2949 (4)	0.6764 (4)	0.2220 (3)	0.0241 (12)	
C6	0.2880 (4)	0.5900 (4)	0.2410 (3)	0.0261 (13)	
C7	0.2118 (4)	0.5517 (4)	0.2454 (3)	0.0283 (13)	
C8	0.2074 (4)	0.4676 (4)	0.2633 (3)	0.0267 (13)	
N2	0.2760 (3)	0.1661 (3)	0.3335 (2)	0.0306 (12)	
N3	0.1395 (4)	0.5897 (4)	0.2351 (3)	0.0376 (14)	
C1	0.2411 (4)	0.7987 (4)	0.1736 (3)	0.0274 (13)	
H1	0.197035	0.825426	0.151745	0.033*	
C13	0.2071 (4)	0.2834 (4)	0.2902 (3)	0.0352 (16)	
H13	0.158167	0.306650	0.272145	0.042*	
C2	0.2315 (4)	0.7178 (4)	0.1898 (3)	0.0288 (14)	
H2	0.181635	0.690235	0.179053	0.035*	
C9	0.2788 (4)	0.4183 (4)	0.2780 (2)	0.0238 (12)	
C18	0.0014 (4)	0.7449 (5)	0.2989 (3)	0.0332 (15)	
H18A	−0.050727	0.774508	0.294198	0.040*	
H18B	−0.009193	0.685479	0.290518	0.040*	
C10	0.3494 (4)	0.4573 (4)	0.2725 (3)	0.0303 (14)	
H10	0.398169	0.426898	0.280828	0.036*	
C43	0.7160 (8)	0.6677 (9)	0.5363 (11)	0.042 (4)	0.755 (6)
H43A	0.750552	0.620589	0.529118	0.051*	0.755 (6)
H43B	0.706612	0.663026	0.574312	0.051*	0.755 (6)
C14	0.2770 (4)	0.3306 (4)	0.2976 (3)	0.0242 (12)	
C5	0.3718 (4)	0.8013 (4)	0.2182 (3)	0.0321 (15)	
H5	0.420999	0.830335	0.228467	0.039*	
C16	0.3437 (4)	0.2111 (4)	0.3409 (3)	0.0307 (14)	
H16	0.391742	0.186128	0.359026	0.037*	
C12	0.2086 (4)	0.2031 (5)	0.3090 (3)	0.0394 (17)	
H12	0.159769	0.172596	0.304362	0.047*	
C19	0.0552 (4)	0.7813 (5)	0.2605 (3)	0.0337 (15)	
H19A	0.065799	0.840471	0.269632	0.040*	
H19B	0.107440	0.751833	0.266156	0.040*	
C11	0.3535 (4)	0.5405 (4)	0.2550 (3)	0.0298 (14)	
H11	0.404999	0.563072	0.252911	0.036*	
C17	0.0445 (4)	0.7544 (5)	0.3571 (3)	0.0356 (15)	
H17A	0.094180	0.720646	0.361516	0.043*	
H17B	0.060774	0.813166	0.362915	0.043*	
C15	0.3468 (4)	0.2913 (4)	0.3237 (3)	0.0307 (14)	
H15	0.396430	0.320484	0.329397	0.037*	
C4	0.3663 (4)	0.7207 (4)	0.2352 (3)	0.0303 (14)	
H4	0.411645	0.695019	0.256283	0.036*	
C21	−0.0251 (4)	0.6369 (6)	0.3971 (3)	0.0514 (18)	
H21A	−0.057542	0.623824	0.425754	0.062*	
H21B	−0.059518	0.626756	0.361612	0.062*	
C20	0.0177 (4)	0.7745 (5)	0.2014 (3)	0.0393 (17)	
H20A	0.055186	0.796317	0.178581	0.059*	
H20B	−0.032317	0.806606	0.194984	0.059*	
H20C	0.006035	0.716167	0.192237	0.059*	
C22	0.0445 (5)	0.5777 (6)	0.4026 (4)	0.054 (2)	
H22A	0.083177	0.591357	0.435677	0.064*	
H22B	0.072677	0.583591	0.370854	0.064*	
C45	0.4409 (5)	0.7848 (5)	0.5039 (4)	0.0272 (17)	0.755 (6)
H45A	0.472410	0.836657	0.510810	0.033*	0.755 (6)
H45B	0.392702	0.797692	0.476886	0.033*	0.755 (6)
C42	0.6356 (6)	0.6606 (6)	0.4986 (4)	0.035 (2)	0.755 (6)
H42A	0.617350	0.602193	0.497120	0.042*	0.755 (6)
H42B	0.642173	0.677743	0.461455	0.042*	0.755 (6)
C33	0.5073 (5)	0.7549 (5)	0.4246 (3)	0.0237 (16)	0.755 (6)
H33A	0.532987	0.710337	0.406125	0.028*	0.755 (6)
H33B	0.454963	0.767687	0.401889	0.028*	0.755 (6)
C25	−0.0828 (4)	0.7757 (7)	0.3948 (4)	0.056 (2)	
H25A	−0.116556	0.757652	0.360633	0.068*	
H25B	−0.110940	0.759399	0.425130	0.068*	
C41	0.5726 (5)	0.7153 (6)	0.5186 (3)	0.0278 (18)	0.755 (6)
H41A	0.595224	0.771764	0.525426	0.033*	0.755 (6)
H41B	0.561719	0.693129	0.553729	0.033*	0.755 (6)
C35	0.5498 (9)	0.8688 (10)	0.3686 (6)	0.041 (3)	0.755 (6)
H35A	0.564860	0.827351	0.342733	0.049*	0.755 (6)
H35B	0.493248	0.885612	0.356326	0.049*	0.755 (6)
C24	0.0809 (6)	0.4250 (7)	0.4101 (4)	0.069 (3)	
H24A	0.105157	0.425932	0.376980	0.104*	
H24B	0.057998	0.369926	0.414501	0.104*	
H24C	0.122096	0.437174	0.441566	0.104*	
C39	0.3383 (7)	0.5482 (8)	0.4330 (6)	0.032 (3)	0.755 (6)
H39A	0.331308	0.527264	0.469252	0.038*	0.755 (6)
H39B	0.375492	0.510520	0.417866	0.038*	0.755 (6)
C47	0.3605 (15)	0.8248 (16)	0.5747 (11)	0.063 (4)	0.755 (6)
H47A	0.353202	0.811780	0.612407	0.075*	0.755 (6)
H47B	0.389626	0.878299	0.575780	0.075*	0.755 (6)
C29	0.0516 (7)	0.7405 (11)	0.4554 (4)	0.033 (3)	0.61 (2)
H29A	0.103397	0.712642	0.453248	0.039*	0.61 (2)
H29B	0.062925	0.800444	0.461057	0.039*	0.61 (2)
C44	0.7608 (8)	0.7487 (9)	0.5299 (6)	0.054 (3)	0.755 (6)
H44A	0.769026	0.754760	0.492094	0.081*	0.755 (6)
H44B	0.813112	0.747672	0.553747	0.081*	0.755 (6)
H44C	0.729072	0.795551	0.539832	0.081*	0.755 (6)
C23	0.0157 (6)	0.4889 (7)	0.4061 (5)	0.077 (3)	
H23A	−0.011659	0.483770	0.438213	0.092*	
H23B	−0.024613	0.476918	0.373488	0.092*	
C46	0.4135 (7)	0.7586 (7)	0.5563 (4)	0.045 (2)	0.755 (6)
H46A	0.383240	0.705812	0.550530	0.054*	0.755 (6)
H46B	0.460903	0.749387	0.584692	0.054*	0.755 (6)
C37	0.4543 (5)	0.6372 (5)	0.4730 (3)	0.0260 (17)	0.755 (6)
H37A	0.490584	0.599457	0.457001	0.031*	0.755 (6)
H37B	0.449155	0.615674	0.509415	0.031*	0.755 (6)
C38	0.3734 (6)	0.6364 (6)	0.4381 (4)	0.035 (2)	0.755 (6)
H38A	0.378051	0.657761	0.401526	0.042*	0.755 (6)
H38B	0.336512	0.673355	0.454088	0.042*	0.755 (6)
C34	0.5595 (6)	0.8310 (6)	0.4271 (4)	0.0323 (19)	0.755 (6)
H34A	0.616476	0.816113	0.439884	0.039*	0.755 (6)
H34B	0.542793	0.871945	0.452742	0.039*	0.755 (6)
C40	0.2563 (9)	0.5506 (10)	0.3951 (9)	0.053 (4)	0.755 (6)
H40A	0.262591	0.577563	0.360841	0.079*	0.755 (6)
H40B	0.217679	0.581988	0.412635	0.079*	0.755 (6)
H40C	0.236540	0.493903	0.387881	0.079*	0.755 (6)
C36	0.6052 (7)	0.9441 (8)	0.3713 (5)	0.056 (3)	0.755 (6)
H36A	0.600722	0.969292	0.335217	0.084*	0.755 (6)
H36B	0.660873	0.926645	0.383600	0.084*	0.755 (6)
H36C	0.589479	0.984706	0.396934	0.084*	0.755 (6)
C30	0.0192 (7)	0.7069 (12)	0.5050 (5)	0.042 (3)	0.61 (2)
H30A	−0.038058	0.722773	0.502717	0.050*	0.61 (2)
H30B	0.022484	0.645609	0.505351	0.050*	0.61 (2)
C27	−0.1596 (7)	0.9090 (9)	0.3871 (5)	0.078 (3)	
H27A	−0.157621	0.962342	0.367618	0.093*	0.59 (2)
H27B	−0.199127	0.872735	0.364587	0.093*	0.59 (2)
H27C	−0.185041	0.902349	0.348684	0.093*	0.41 (2)
H27D	−0.193026	0.878323	0.409611	0.093*	0.41 (2)
C31	0.0672 (10)	0.7413 (13)	0.5569 (5)	0.047 (4)	0.61 (2)
H31A	0.055774	0.708577	0.588334	0.057*	0.61 (2)
H31B	0.125460	0.736824	0.555375	0.057*	0.61 (2)
C26	−0.0765 (7)	0.8680 (9)	0.3942 (6)	0.089 (4)	
H26A	−0.048299	0.885228	0.364137	0.107*	
H26B	−0.044150	0.887091	0.428730	0.107*	
C32	0.0458 (15)	0.8288 (15)	0.5637 (7)	0.073 (7)	0.61 (2)
H32A	0.064517	0.862366	0.535403	0.109*	0.61 (2)
H32B	0.071469	0.848351	0.599510	0.109*	0.61 (2)
H32C	−0.012693	0.833890	0.560626	0.109*	0.61 (2)
C28	−0.1850 (19)	0.9237 (14)	0.4371 (10)	0.107 (9)	0.59 (2)
H28A	−0.192302	0.870573	0.454883	0.161*	0.59 (2)
H28B	−0.236140	0.953935	0.431105	0.161*	0.59 (2)
H28C	−0.144361	0.956675	0.460395	0.161*	0.59 (2)
C28A	−0.1618 (13)	0.9912 (14)	0.4001 (13)	0.073 (8)	0.41 (2)
H28D	−0.147673	0.997932	0.439495	0.110*	0.41 (2)
H28E	−0.216065	1.012963	0.387951	0.110*	0.41 (2)
H28F	−0.123226	1.021782	0.382040	0.110*	0.41 (2)
I4A	0.2790 (4)	1.1720 (5)	0.1343 (6)	0.071 (2)	0.335 (11)
I5A	0.2107 (2)	0.9352 (7)	0.05393 (15)	0.0444 (12)	0.335 (11)
C48	0.2807 (11)	0.8366 (14)	0.5423 (10)	0.123 (8)	0.755 (6)
H48A	0.286474	0.857461	0.506225	0.184*	0.755 (6)
H48B	0.250234	0.876771	0.560401	0.184*	0.755 (6)
H48C	0.252004	0.783513	0.538606	0.184*	0.755 (6)
C29A	0.0426 (16)	0.773 (2)	0.4518 (7)	0.054 (6)	0.39 (2)
H29C	0.098885	0.753067	0.457262	0.065*	0.39 (2)
H29D	0.043745	0.833305	0.444194	0.065*	0.39 (2)
C30A	0.0098 (17)	0.761 (3)	0.5048 (8)	0.067 (6)	0.39 (2)
H30C	−0.037872	0.797006	0.504690	0.080*	0.39 (2)
H30D	−0.007561	0.702698	0.507415	0.080*	0.39 (2)
C31A	0.072 (2)	0.782 (2)	0.5535 (9)	0.065 (7)	0.39 (2)
H31C	0.053828	0.760840	0.586792	0.078*	0.39 (2)
H31D	0.123214	0.753321	0.550092	0.078*	0.39 (2)
C32A	0.087 (3)	0.871 (2)	0.5588 (13)	0.080 (9)	0.39 (2)
H32D	0.118279	0.889632	0.531276	0.120*	0.39 (2)
H32E	0.116315	0.883584	0.595092	0.120*	0.39 (2)
H32F	0.035066	0.900805	0.553676	0.120*	0.39 (2)
C37A	0.4269 (13)	0.6684 (12)	0.4414 (8)	0.024 (4)	0.245 (6)
H37C	0.379856	0.704346	0.428985	0.028*	0.245 (6)
H37D	0.449952	0.652430	0.408808	0.028*	0.245 (6)
C38A	0.3973 (15)	0.5919 (13)	0.4647 (10)	0.031 (5)	0.245 (6)
H38C	0.365249	0.607594	0.493116	0.038*	0.245 (6)
H38D	0.444043	0.559148	0.482374	0.038*	0.245 (6)
C39A	0.346 (2)	0.537 (2)	0.422 (2)	0.038 (9)	0.245 (6)
H39C	0.333660	0.484185	0.439250	0.046*	0.245 (6)
H39D	0.375304	0.525267	0.392201	0.046*	0.245 (6)
C40A	0.267 (2)	0.583 (3)	0.401 (2)	0.050 (11)	0.245 (6)
H40D	0.273317	0.614878	0.368257	0.075*	0.245 (6)
H40E	0.252447	0.620134	0.428531	0.075*	0.245 (6)
H40F	0.223428	0.541917	0.390907	0.075*	0.245 (6)
C33A	0.4946 (16)	0.7987 (14)	0.4441 (10)	0.033 (4)	0.245 (6)
H33C	0.439813	0.822860	0.436963	0.040*	0.245 (6)
H33D	0.529872	0.839840	0.465704	0.040*	0.245 (6)
C34A	0.5233 (18)	0.7885 (18)	0.3903 (10)	0.042 (5)	0.245 (6)
H34C	0.477423	0.778029	0.360943	0.051*	0.245 (6)
H34D	0.561496	0.741493	0.391721	0.051*	0.245 (6)
C35A	0.566 (4)	0.872 (3)	0.3804 (16)	0.043 (8)	0.245 (6)
H35C	0.527382	0.918353	0.380606	0.052*	0.245 (6)
H35D	0.611400	0.881369	0.410231	0.052*	0.245 (6)
C36A	0.597 (3)	0.871 (3)	0.3269 (17)	0.090 (13)	0.245 (6)
H36D	0.551983	0.881733	0.297350	0.136*	0.245 (6)
H36E	0.619708	0.816150	0.321558	0.136*	0.245 (6)
H36F	0.637954	0.913489	0.327029	0.136*	0.245 (6)
C41A	0.5670 (10)	0.6710 (13)	0.4886 (11)	0.028 (4)	0.245 (6)
H41C	0.575487	0.643749	0.454441	0.033*	0.245 (6)
H41D	0.562258	0.627342	0.515858	0.033*	0.245 (6)
C42A	0.6377 (12)	0.7274 (14)	0.5091 (12)	0.031 (5)	0.245 (6)
H42C	0.625197	0.761513	0.539679	0.037*	0.245 (6)
H42D	0.647779	0.765259	0.479647	0.037*	0.245 (6)
C43A	0.7122 (17)	0.675 (2)	0.528 (3)	0.039 (8)	0.245 (6)
H43C	0.721926	0.638239	0.497724	0.046*	0.245 (6)
H43D	0.702406	0.639280	0.558434	0.046*	0.245 (6)
C44A	0.7869 (18)	0.728 (2)	0.5459 (15)	0.039 (7)	0.245 (6)
H44D	0.810451	0.744302	0.514045	0.059*	0.245 (6)
H44E	0.826364	0.695707	0.570715	0.059*	0.245 (6)
H44F	0.771891	0.777574	0.564568	0.059*	0.245 (6)
C45A	0.4547 (16)	0.746 (2)	0.5276 (9)	0.038 (5)	0.245 (6)
H45C	0.492477	0.784397	0.549689	0.046*	0.245 (6)
H45D	0.451957	0.695197	0.549589	0.046*	0.245 (6)
C46A	0.3728 (17)	0.786 (2)	0.5196 (13)	0.052 (6)	0.245 (6)
H46C	0.332280	0.744564	0.503243	0.062*	0.245 (6)
H46D	0.371980	0.832499	0.493889	0.062*	0.245 (6)
C47A	0.350 (4)	0.817 (5)	0.572 (3)	0.055 (9)	0.245 (6)
H47C	0.294106	0.837339	0.563400	0.066*	0.245 (6)
H47D	0.349257	0.768176	0.596017	0.066*	0.245 (6)
C48A	0.397 (3)	0.881 (3)	0.6041 (16)	0.068 (10)	0.245 (6)
H48D	0.420348	0.918769	0.579832	0.102*	0.245 (6)
H48E	0.440020	0.855692	0.629757	0.102*	0.245 (6)
H48F	0.361612	0.913132	0.624149	0.102*	0.245 (6)

### catena-Poly[[triiodidobismuth(III)]-di-µ-iodido-[triiodidobismuth(III)]-µ-4,7-bis(pyridin-4-yl)benzo[c][1,2,5]thiadiazole] Atomic displacement parameters (Å^2^)

**Table:**

	*U*^11^	*U*^22^	*U*^33^	*U*^12^	*U*^13^	*U*^23^
Bi2	0.01765 (11)	0.02322 (12)	0.02434 (12)	−0.00012 (8)	0.00231 (8)	0.00378 (8)
Bi1	0.01542 (11)	0.03014 (13)	0.02640 (13)	0.00398 (8)	0.00543 (9)	0.01177 (9)
I2	0.01511 (17)	0.0329 (2)	0.02471 (19)	0.00083 (14)	0.00270 (14)	0.00730 (15)
I3	0.01726 (17)	0.0295 (2)	0.02784 (19)	0.00128 (14)	0.00703 (14)	0.00341 (15)
I7	0.02368 (19)	0.0240 (2)	0.0368 (2)	−0.00310 (14)	−0.00340 (15)	0.00517 (16)
I1	0.01724 (17)	0.0402 (2)	0.0301 (2)	−0.00221 (16)	0.00331 (14)	−0.00063 (17)
I6	0.0406 (2)	0.0277 (2)	0.0260 (2)	−0.00350 (17)	−0.00720 (16)	0.00053 (16)
I8	0.0342 (2)	0.0592 (3)	0.0563 (3)	−0.0025 (2)	0.0242 (2)	−0.0063 (3)
I5	0.0211 (5)	0.0452 (9)	0.0215 (5)	−0.0047 (5)	0.0028 (3)	0.0070 (5)
I4	0.0321 (7)	0.0220 (4)	0.0409 (8)	0.0038 (4)	0.0158 (4)	0.0081 (4)
S1	0.0204 (8)	0.0320 (9)	0.0891 (16)	0.0038 (7)	0.0186 (9)	0.0186 (10)
N4	0.022 (3)	0.028 (3)	0.060 (4)	0.004 (2)	0.013 (3)	0.010 (3)
N6	0.032 (3)	0.021 (2)	0.025 (2)	0.0059 (19)	0.0065 (19)	−0.0009 (19)
N1	0.020 (2)	0.026 (3)	0.042 (3)	0.001 (2)	0.005 (2)	0.006 (2)
N5	0.021 (3)	0.088 (4)	0.032 (3)	−0.011 (3)	0.001 (2)	0.009 (3)
C3	0.019 (3)	0.027 (3)	0.027 (3)	0.002 (2)	0.007 (2)	0.005 (2)
C6	0.024 (3)	0.026 (3)	0.028 (3)	−0.003 (2)	0.006 (2)	0.000 (3)
C7	0.024 (3)	0.025 (3)	0.038 (4)	0.001 (2)	0.010 (3)	0.004 (3)
C8	0.020 (3)	0.030 (3)	0.030 (3)	−0.001 (2)	0.004 (2)	0.005 (3)
N2	0.025 (3)	0.023 (3)	0.043 (3)	0.000 (2)	0.003 (2)	0.000 (2)
N3	0.025 (3)	0.029 (3)	0.060 (4)	0.002 (2)	0.013 (3)	0.009 (3)
C1	0.024 (3)	0.028 (3)	0.029 (3)	0.005 (2)	0.002 (2)	0.003 (3)
C13	0.022 (3)	0.028 (3)	0.052 (4)	0.000 (3)	−0.007 (3)	0.012 (3)
C2	0.021 (3)	0.037 (4)	0.028 (3)	−0.001 (3)	0.002 (2)	0.004 (3)
C9	0.021 (3)	0.025 (3)	0.025 (3)	0.001 (2)	0.002 (2)	0.003 (2)
C18	0.025 (3)	0.044 (4)	0.030 (3)	−0.002 (3)	0.001 (2)	0.013 (3)
C10	0.019 (3)	0.026 (3)	0.043 (4)	0.001 (2)	−0.002 (3)	0.005 (3)
C43	0.031 (5)	0.052 (6)	0.039 (9)	0.021 (4)	−0.009 (5)	−0.003 (5)
C14	0.024 (3)	0.021 (3)	0.026 (3)	0.001 (2)	0.000 (2)	−0.001 (2)
C5	0.020 (3)	0.028 (3)	0.048 (4)	0.000 (3)	0.004 (3)	0.005 (3)
C16	0.024 (3)	0.030 (3)	0.034 (3)	0.003 (3)	−0.004 (3)	0.002 (3)
C12	0.024 (3)	0.030 (4)	0.062 (5)	−0.001 (3)	−0.001 (3)	−0.001 (3)
C19	0.027 (3)	0.042 (4)	0.033 (3)	−0.002 (3)	0.007 (3)	0.007 (3)
C11	0.019 (3)	0.031 (3)	0.039 (4)	−0.001 (3)	0.005 (3)	0.004 (3)
C17	0.022 (3)	0.051 (4)	0.032 (3)	−0.011 (3)	0.002 (2)	0.009 (3)
C15	0.021 (3)	0.032 (3)	0.036 (3)	0.000 (3)	−0.004 (3)	0.004 (3)
C4	0.020 (3)	0.030 (3)	0.041 (4)	0.002 (3)	0.005 (3)	0.007 (3)
C21	0.025 (3)	0.092 (5)	0.034 (4)	−0.015 (3)	−0.004 (3)	0.027 (4)
C20	0.032 (4)	0.056 (5)	0.031 (3)	0.012 (3)	0.007 (3)	0.010 (3)
C22	0.028 (4)	0.085 (5)	0.043 (4)	−0.018 (3)	−0.006 (3)	0.029 (4)
C45	0.030 (4)	0.022 (4)	0.031 (4)	0.009 (3)	0.009 (3)	−0.001 (3)
C42	0.038 (4)	0.039 (5)	0.026 (4)	0.019 (4)	−0.002 (3)	−0.007 (4)
C33	0.027 (4)	0.027 (4)	0.016 (3)	0.001 (3)	0.001 (3)	0.002 (3)
C25	0.020 (3)	0.105 (6)	0.043 (4)	−0.002 (4)	0.000 (3)	0.005 (5)
C41	0.029 (4)	0.030 (4)	0.022 (4)	0.009 (3)	0.000 (3)	0.000 (3)
C35	0.038 (7)	0.047 (6)	0.039 (7)	0.010 (5)	0.013 (6)	0.023 (5)
C24	0.066 (6)	0.078 (6)	0.051 (5)	−0.027 (4)	−0.029 (4)	0.031 (5)
C39	0.027 (4)	0.030 (5)	0.045 (6)	0.001 (4)	0.028 (4)	−0.003 (4)
C47	0.086 (9)	0.045 (8)	0.070 (8)	0.016 (7)	0.051 (7)	−0.002 (7)
C29	0.018 (5)	0.060 (8)	0.021 (4)	0.015 (5)	0.003 (3)	0.019 (5)
C44	0.032 (6)	0.070 (8)	0.057 (9)	0.014 (5)	−0.001 (5)	−0.010 (7)
C23	0.046 (5)	0.094 (6)	0.077 (7)	−0.031 (4)	−0.029 (5)	0.053 (6)
C46	0.059 (6)	0.043 (6)	0.037 (5)	0.015 (5)	0.023 (4)	0.001 (4)
C37	0.034 (4)	0.014 (3)	0.029 (4)	0.008 (3)	0.004 (3)	0.003 (3)
C38	0.042 (5)	0.026 (4)	0.035 (5)	0.004 (4)	0.001 (4)	−0.002 (4)
C34	0.031 (4)	0.036 (4)	0.029 (4)	−0.001 (3)	0.004 (3)	0.004 (3)
C40	0.044 (6)	0.061 (10)	0.053 (7)	−0.017 (7)	0.007 (5)	−0.010 (8)
C36	0.043 (6)	0.068 (7)	0.057 (7)	−0.010 (5)	0.006 (5)	0.026 (6)
C30	0.030 (6)	0.069 (9)	0.028 (5)	0.008 (6)	0.010 (4)	0.011 (5)
C27	0.054 (6)	0.109 (8)	0.073 (7)	0.011 (5)	0.018 (5)	−0.005 (6)
C31	0.060 (8)	0.059 (10)	0.024 (5)	0.012 (7)	0.012 (5)	0.008 (6)
C26	0.041 (5)	0.113 (7)	0.108 (9)	0.004 (5)	−0.007 (5)	−0.033 (8)
C32	0.100 (15)	0.073 (12)	0.039 (8)	0.028 (11)	−0.011 (9)	−0.011 (8)
C28	0.17 (2)	0.064 (13)	0.106 (14)	0.002 (13)	0.088 (16)	0.006 (11)
C28A	0.022 (10)	0.097 (10)	0.10 (2)	0.003 (9)	−0.001 (11)	0.011 (12)
I4A	0.0351 (17)	0.044 (2)	0.140 (6)	0.0164 (15)	0.032 (3)	0.053 (3)
I5A	0.0220 (8)	0.086 (4)	0.0238 (8)	0.0083 (16)	0.0010 (5)	−0.0027 (16)
C48	0.074 (9)	0.143 (17)	0.165 (17)	0.052 (10)	0.061 (10)	0.083 (14)
C29A	0.031 (10)	0.097 (15)	0.030 (6)	−0.010 (11)	−0.006 (6)	0.014 (8)
C30A	0.075 (12)	0.086 (15)	0.041 (7)	−0.023 (12)	0.016 (8)	0.008 (10)
C31A	0.093 (14)	0.067 (15)	0.034 (8)	−0.008 (14)	0.006 (9)	0.019 (12)
C32A	0.11 (2)	0.071 (16)	0.061 (17)	−0.008 (15)	0.009 (16)	−0.011 (13)
C37A	0.023 (8)	0.026 (8)	0.021 (9)	0.010 (6)	−0.002 (7)	0.008 (7)
C38A	0.029 (10)	0.022 (9)	0.044 (11)	0.006 (7)	0.008 (8)	0.002 (8)
C39A	0.034 (13)	0.036 (12)	0.047 (16)	−0.003 (9)	0.013 (12)	−0.007 (11)
C40A	0.039 (15)	0.06 (3)	0.05 (2)	0.002 (17)	0.007 (12)	0.01 (2)
C33A	0.024 (10)	0.034 (9)	0.039 (9)	0.003 (7)	−0.002 (8)	0.014 (8)
C34A	0.034 (11)	0.051 (12)	0.042 (10)	0.011 (9)	0.006 (9)	0.025 (9)
C35A	0.040 (17)	0.054 (13)	0.033 (15)	0.002 (12)	−0.001 (13)	0.009 (12)
C36A	0.10 (3)	0.11 (3)	0.073 (19)	−0.01 (2)	0.050 (19)	0.03 (2)
C41A	0.026 (7)	0.017 (8)	0.037 (11)	0.002 (6)	−0.002 (7)	−0.007 (8)
C42A	0.026 (7)	0.022 (10)	0.044 (12)	−0.004 (7)	0.000 (8)	−0.008 (9)
C43A	0.031 (9)	0.050 (15)	0.035 (18)	0.012 (9)	0.003 (10)	−0.012 (13)
C44A	0.030 (11)	0.048 (17)	0.033 (16)	0.015 (12)	−0.014 (12)	0.004 (13)
C45A	0.042 (11)	0.043 (13)	0.031 (9)	0.006 (9)	0.012 (8)	−0.004 (8)
C46A	0.052 (11)	0.046 (13)	0.060 (12)	0.018 (10)	0.021 (10)	−0.006 (11)
C47A	0.061 (16)	0.050 (17)	0.062 (14)	0.007 (13)	0.034 (13)	−0.004 (13)
C48A	0.09 (2)	0.06 (2)	0.066 (19)	−0.018 (18)	0.053 (17)	−0.010 (15)

### catena-Poly[[triiodidobismuth(III)]-di-µ-iodido-[triiodidobismuth(III)]-µ-4,7-bis(pyridin-4-yl)benzo[c][1,2,5]thiadiazole] Geometric parameters (Å, º)

**Table:**

Bi2—I2^i^	3.2756 (4)	C47—C48	1.46 (4)
Bi2—I7	2.9571 (5)	C29—H29A	0.9900
Bi2—I1^i^	3.2267 (4)	C29—H29B	0.9900
Bi2—I6	2.9290 (5)	C29—C30	1.529 (14)
Bi2—I8	2.9489 (5)	C44—H44A	0.9800
Bi2—N2	2.671 (6)	C44—H44B	0.9800
Bi1—I2	3.1757 (4)	C44—H44C	0.9800
Bi1—I3	2.9840 (4)	C23—H23A	0.9900
Bi1—I1	3.1292 (5)	C23—H23B	0.9900
Bi1—I5	2.9657 (19)	C46—H46A	0.9900
Bi1—I4	3.0143 (13)	C46—H46B	0.9900
Bi1—N1	2.655 (5)	C37—H37A	0.9900
Bi1—I4A	2.859 (4)	C37—H37B	0.9900
Bi1—I5A	3.131 (5)	C37—C38	1.494 (12)
S1—N4	1.617 (6)	C38—H38A	0.9900
S1—N3	1.620 (6)	C38—H38B	0.9900
N4—C8	1.352 (8)	C34—H34A	0.9900
N6—C45	1.518 (9)	C34—H34B	0.9900
N6—C33	1.518 (9)	C40—H40A	0.9800
N6—C41	1.557 (9)	C40—H40B	0.9800
N6—C37	1.502 (9)	C40—H40C	0.9800
N6—C37A	1.574 (17)	C36—H36A	0.9800
N6—C33A	1.516 (17)	C36—H36B	0.9800
N6—C41A	1.501 (17)	C36—H36C	0.9800
N6—C45A	1.498 (17)	C30—H30A	0.9900
N1—C1	1.337 (9)	C30—H30B	0.9900
N1—C5	1.356 (9)	C30—C31	1.517 (17)
N5—C17	1.517 (9)	C27—H27A	0.9900
N5—C21	1.535 (13)	C27—H27B	0.9900
N5—C25	1.511 (11)	C27—H27C	0.9900
N5—C29	1.530 (12)	C27—H27D	0.9900
N5—C29A	1.547 (17)	C27—C26	1.533 (15)
C3—C6	1.481 (9)	C27—C28	1.404 (19)
C3—C2	1.397 (9)	C27—C28A	1.37 (2)
C3—C4	1.391 (9)	C31—H31A	0.9900
C6—C7	1.445 (9)	C31—H31B	0.9900
C6—C11	1.361 (9)	C31—C32	1.47 (2)
C7—C8	1.433 (9)	C26—H26A	0.9900
C7—N3	1.351 (9)	C26—H26B	0.9900
C8—C9	1.439 (9)	C32—H32A	0.9800
N2—C16	1.338 (9)	C32—H32B	0.9800
N2—C12	1.340 (9)	C32—H32C	0.9800
C1—H1	0.9500	C28—H28A	0.9800
C1—C2	1.383 (10)	C28—H28B	0.9800
C13—H13	0.9500	C28—H28C	0.9800
C13—C14	1.389 (9)	C28A—H28D	0.9800
C13—C12	1.375 (10)	C28A—H28E	0.9800
C2—H2	0.9500	C28A—H28F	0.9800
C9—C10	1.372 (9)	C48—H48A	0.9800
C9—C14	1.497 (9)	C48—H48B	0.9800
C18—H18A	0.9900	C48—H48C	0.9800
C18—H18B	0.9900	C29A—H29C	0.9900
C18—C19	1.538 (9)	C29A—H29D	0.9900
C18—C17	1.521 (9)	C29A—C30A	1.525 (18)
C10—H10	0.9500	C30A—H30C	0.9900
C10—C11	1.414 (10)	C30A—H30D	0.9900
C43—H43A	0.9900	C30A—C31A	1.51 (2)
C43—H43B	0.9900	C31A—H31C	0.9900
C43—C42	1.522 (14)	C31A—H31D	0.9900
C43—C44	1.529 (17)	C31A—C32A	1.47 (3)
C14—C15	1.400 (9)	C32A—H32D	0.9800
C5—H5	0.9500	C32A—H32E	0.9800
C5—C4	1.375 (9)	C32A—H32F	0.9800
C16—H16	0.9500	C37A—H37C	0.9900
C16—C15	1.366 (10)	C37A—H37D	0.9900
C12—H12	0.9500	C37A—C38A	1.484 (19)
C19—H19A	0.9900	C38A—H38C	0.9900
C19—H19B	0.9900	C38A—H38D	0.9900
C19—C20	1.511 (10)	C38A—C39A	1.53 (2)
C11—H11	0.9500	C39A—H39C	0.9900
C17—H17A	0.9900	C39A—H39D	0.9900
C17—H17B	0.9900	C39A—C40A	1.54 (2)
C15—H15	0.9500	C40A—H40D	0.9800
C4—H4	0.9500	C40A—H40E	0.9800
C21—H21A	0.9900	C40A—H40F	0.9800
C21—H21B	0.9900	C33A—H33C	0.9900
C21—C22	1.501 (13)	C33A—H33D	0.9900
C20—H20A	0.9800	C33A—C34A	1.51 (2)
C20—H20B	0.9800	C34A—H34C	0.9900
C20—H20C	0.9800	C34A—H34D	0.9900
C22—H22A	0.9900	C34A—C35A	1.56 (2)
C22—H22B	0.9900	C35A—H35C	0.9900
C22—C23	1.520 (14)	C35A—H35D	0.9900
C45—H45A	0.9900	C35A—C36A	1.51 (3)
C45—H45B	0.9900	C36A—H36D	0.9800
C45—C46	1.515 (12)	C36A—H36E	0.9800
C42—H42A	0.9900	C36A—H36F	0.9800
C42—H42B	0.9900	C41A—H41C	0.9900
C42—C41	1.524 (11)	C41A—H41D	0.9900
C33—H33A	0.9900	C41A—C42A	1.520 (19)
C33—H33B	0.9900	C42A—H42C	0.9900
C33—C34	1.505 (12)	C42A—H42D	0.9900
C25—H25A	0.9900	C42A—C43A	1.52 (2)
C25—H25B	0.9900	C43A—H43C	0.9900
C25—C26	1.492 (17)	C43A—H43D	0.9900
C41—H41A	0.9900	C43A—C44A	1.53 (2)
C41—H41B	0.9900	C44A—H44D	0.9800
C35—H35A	0.9900	C44A—H44E	0.9800
C35—H35B	0.9900	C44A—H44F	0.9800
C35—C34	1.565 (14)	C45A—H45C	0.9900
C35—C36	1.526 (18)	C45A—H45D	0.9900
C24—H24A	0.9800	C45A—C46A	1.50 (2)
C24—H24B	0.9800	C46A—H46C	0.9900
C24—H24C	0.9800	C46A—H46D	0.9900
C24—C23	1.497 (17)	C46A—C47A	1.50 (3)
C39—H39A	0.9900	C47A—H47C	0.9900
C39—H39B	0.9900	C47A—H47D	0.9900
C39—C38	1.536 (14)	C47A—C48A	1.46 (4)
C39—C40	1.543 (15)	C48A—H48D	0.9800
C47—H47A	0.9900	C48A—H48E	0.9800
C47—H47B	0.9900	C48A—H48F	0.9800
C47—C46	1.510 (19)		
			
I7—Bi2—I2^i^	92.778 (12)	C43—C44—H44B	109.5
I7—Bi2—I1^i^	97.092 (13)	C43—C44—H44C	109.5
I1^i^—Bi2—I2^i^	83.901 (11)	H44A—C44—H44B	109.5
I6—Bi2—I2^i^	170.327 (14)	H44A—C44—H44C	109.5
I6—Bi2—I7	94.290 (13)	H44B—C44—H44C	109.5
I6—Bi2—I1^i^	88.675 (13)	C22—C23—H23A	108.6
I6—Bi2—I8	97.771 (17)	C22—C23—H23B	108.6
I8—Bi2—I2^i^	88.211 (15)	C24—C23—C22	114.5 (8)
I8—Bi2—I7	94.710 (16)	C24—C23—H23A	108.6
I8—Bi2—I1^i^	166.092 (17)	C24—C23—H23B	108.6
N2—Bi2—I2^i^	84.97 (13)	H23A—C23—H23B	107.6
N2—Bi2—I7	174.95 (13)	C45—C46—H46A	109.6
N2—Bi2—I1^i^	78.19 (12)	C45—C46—H46B	109.6
N2—Bi2—I6	87.45 (13)	C47—C46—C45	110.1 (14)
N2—Bi2—I8	89.75 (12)	C47—C46—H46A	109.6
I3—Bi1—I2	167.787 (13)	C47—C46—H46B	109.6
I3—Bi1—I1	85.099 (12)	H46A—C46—H46B	108.2
I3—Bi1—I4	98.12 (4)	N6—C37—H37A	109.0
I3—Bi1—I5A	89.26 (7)	N6—C37—H37B	109.0
I1—Bi1—I2	87.168 (12)	H37A—C37—H37B	107.8
I1—Bi1—I5A	165.37 (19)	C38—C37—N6	113.1 (7)
I5—Bi1—I2	93.81 (5)	C38—C37—H37A	109.0
I5—Bi1—I3	93.08 (6)	C38—C37—H37B	109.0
I5—Bi1—I1	174.85 (6)	C39—C38—H38A	109.4
I5—Bi1—I4	96.54 (3)	C39—C38—H38B	109.4
I4—Bi1—I2	91.10 (4)	C37—C38—C39	111.0 (8)
I4—Bi1—I1	88.49 (4)	C37—C38—H38A	109.4
N1—Bi1—I2	83.58 (12)	C37—C38—H38B	109.4
N1—Bi1—I3	86.32 (12)	H38A—C38—H38B	108.0
N1—Bi1—I1	84.81 (13)	C33—C34—C35	107.8 (9)
N1—Bi1—I5	90.27 (14)	C33—C34—H34A	110.2
N1—Bi1—I4	171.63 (13)	C33—C34—H34B	110.2
N1—Bi1—I4A	172.5 (2)	C35—C34—H34A	110.2
N1—Bi1—I5A	81.4 (2)	C35—C34—H34B	110.2
I4A—Bi1—I2	91.29 (10)	H34A—C34—H34B	108.5
I4A—Bi1—I3	99.40 (8)	C39—C40—H40A	109.5
I4A—Bi1—I1	100.4 (3)	C39—C40—H40B	109.5
I4A—Bi1—I5A	93.80 (15)	C39—C40—H40C	109.5
I5A—Bi1—I2	95.97 (7)	H40A—C40—H40B	109.5
Bi1—I2—Bi2^ii^	93.245 (11)	H40A—C40—H40C	109.5
Bi1—I1—Bi2^ii^	95.084 (12)	H40B—C40—H40C	109.5
N4—S1—N3	100.6 (3)	C35—C36—H36A	109.5
C8—N4—S1	106.9 (5)	C35—C36—H36B	109.5
C45—N6—C41	106.2 (6)	C35—C36—H36C	109.5
C33—N6—C45	108.9 (6)	H36A—C36—H36B	109.5
C33—N6—C41	109.2 (6)	H36A—C36—H36C	109.5
C37—N6—C45	113.2 (6)	H36B—C36—H36C	109.5
C37—N6—C33	111.1 (6)	C29—C30—H30A	109.6
C37—N6—C41	108.0 (6)	C29—C30—H30B	109.6
C33A—N6—C37A	101.0 (14)	H30A—C30—H30B	108.1
C41A—N6—C37A	106.3 (12)	C31—C30—C29	110.5 (11)
C41A—N6—C33A	115.7 (15)	C31—C30—H30A	109.6
C45A—N6—C37A	106.6 (16)	C31—C30—H30B	109.6
C45A—N6—C33A	108.2 (17)	H27A—C27—H27B	107.9
C45A—N6—C41A	117.4 (16)	H27C—C27—H27D	107.3
C1—N1—Bi1	116.8 (4)	C26—C27—H27A	109.2
C1—N1—C5	117.4 (6)	C26—C27—H27B	109.2
C5—N1—Bi1	125.1 (4)	C26—C27—H27C	108.1
C17—N5—C21	111.1 (7)	C26—C27—H27D	108.1
C17—N5—C29	106.8 (7)	C28—C27—H27A	109.2
C17—N5—C29A	102.3 (10)	C28—C27—H27B	109.2
C21—N5—C29A	125.0 (14)	C28—C27—C26	111.9 (17)
C25—N5—C17	111.3 (6)	C28A—C27—H27C	108.1
C25—N5—C21	106.2 (6)	C28A—C27—H27D	108.1
C25—N5—C29	115.6 (8)	C28A—C27—C26	116.8 (14)
C25—N5—C29A	100.3 (15)	C30—C31—H31A	109.7
C29—N5—C21	105.7 (8)	C30—C31—H31B	109.7
C2—C3—C6	122.7 (6)	H31A—C31—H31B	108.2
C4—C3—C6	121.1 (6)	C32—C31—C30	110.0 (13)
C4—C3—C2	116.2 (6)	C32—C31—H31A	109.7
C7—C6—C3	122.9 (6)	C32—C31—H31B	109.7
C11—C6—C3	122.1 (6)	C25—C26—C27	111.5 (10)
C11—C6—C7	115.0 (6)	C25—C26—H26A	109.3
C8—C7—C6	121.3 (6)	C25—C26—H26B	109.3
N3—C7—C6	125.2 (6)	C27—C26—H26A	109.3
N3—C7—C8	113.4 (6)	C27—C26—H26B	109.3
N4—C8—C7	112.7 (6)	H26A—C26—H26B	108.0
N4—C8—C9	126.1 (6)	C31—C32—H32A	109.5
C7—C8—C9	121.2 (6)	C31—C32—H32B	109.5
C16—N2—Bi2	124.3 (4)	C31—C32—H32C	109.5
C16—N2—C12	117.5 (6)	H32A—C32—H32B	109.5
C12—N2—Bi2	117.1 (4)	H32A—C32—H32C	109.5
C7—N3—S1	106.4 (5)	H32B—C32—H32C	109.5
N1—C1—H1	118.6	C27—C28—H28A	109.5
N1—C1—C2	122.9 (6)	C27—C28—H28B	109.5
C2—C1—H1	118.6	C27—C28—H28C	109.5
C14—C13—H13	120.0	H28A—C28—H28B	109.5
C12—C13—H13	120.0	H28A—C28—H28C	109.5
C12—C13—C14	120.1 (6)	H28B—C28—H28C	109.5
C3—C2—H2	119.9	C27—C28A—H28D	109.5
C1—C2—C3	120.3 (6)	C27—C28A—H28E	109.5
C1—C2—H2	119.9	C27—C28A—H28F	109.5
C8—C9—C14	122.9 (5)	H28D—C28A—H28E	109.5
C10—C9—C8	115.1 (6)	H28D—C28A—H28F	109.5
C10—C9—C14	121.9 (6)	H28E—C28A—H28F	109.5
H18A—C18—H18B	108.4	C47—C48—H48A	109.5
C19—C18—H18A	110.0	C47—C48—H48B	109.5
C19—C18—H18B	110.0	C47—C48—H48C	109.5
C17—C18—H18A	110.0	H48A—C48—H48B	109.5
C17—C18—H18B	110.0	H48A—C48—H48C	109.5
C17—C18—C19	108.5 (6)	H48B—C48—H48C	109.5
C9—C10—H10	118.3	N5—C29A—H29C	108.1
C9—C10—C11	123.4 (6)	N5—C29A—H29D	108.1
C11—C10—H10	118.3	H29C—C29A—H29D	107.3
H43A—C43—H43B	107.7	C30A—C29A—N5	116.6 (17)
C42—C43—H43A	108.8	C30A—C29A—H29C	108.1
C42—C43—H43B	108.8	C30A—C29A—H29D	108.1
C42—C43—C44	113.9 (12)	C29A—C30A—H30C	109.3
C44—C43—H43A	108.8	C29A—C30A—H30D	109.3
C44—C43—H43B	108.8	H30C—C30A—H30D	108.0
C13—C14—C9	122.3 (6)	C31A—C30A—C29A	111.6 (19)
C13—C14—C15	116.3 (6)	C31A—C30A—H30C	109.3
C15—C14—C9	121.3 (6)	C31A—C30A—H30D	109.3
N1—C5—H5	118.8	C30A—C31A—H31C	109.2
N1—C5—C4	122.5 (6)	C30A—C31A—H31D	109.2
C4—C5—H5	118.8	H31C—C31A—H31D	107.9
N2—C16—H16	118.5	C32A—C31A—C30A	112 (2)
N2—C16—C15	122.9 (6)	C32A—C31A—H31C	109.2
C15—C16—H16	118.5	C32A—C31A—H31D	109.2
N2—C12—C13	122.9 (7)	C31A—C32A—H32D	109.5
N2—C12—H12	118.6	C31A—C32A—H32E	109.5
C13—C12—H12	118.6	C31A—C32A—H32F	109.5
C18—C19—H19A	109.1	H32D—C32A—H32E	109.5
C18—C19—H19B	109.1	H32D—C32A—H32F	109.5
H19A—C19—H19B	107.8	H32E—C32A—H32F	109.5
C20—C19—C18	112.7 (6)	N6—C37A—H37C	107.8
C20—C19—H19A	109.1	N6—C37A—H37D	107.8
C20—C19—H19B	109.1	H37C—C37A—H37D	107.2
C6—C11—C10	123.9 (6)	C38A—C37A—N6	117.9 (15)
C6—C11—H11	118.0	C38A—C37A—H37C	107.8
C10—C11—H11	118.0	C38A—C37A—H37D	107.8
N5—C17—C18	115.6 (5)	C37A—C38A—H38C	108.9
N5—C17—H17A	108.4	C37A—C38A—H38D	108.9
N5—C17—H17B	108.4	C37A—C38A—C39A	113 (2)
C18—C17—H17A	108.4	H38C—C38A—H38D	107.7
C18—C17—H17B	108.4	C39A—C38A—H38C	108.9
H17A—C17—H17B	107.4	C39A—C38A—H38D	108.9
C14—C15—H15	119.9	C38A—C39A—H39C	109.7
C16—C15—C14	120.2 (6)	C38A—C39A—H39D	109.7
C16—C15—H15	119.9	C38A—C39A—C40A	110 (2)
C3—C4—H4	119.6	H39C—C39A—H39D	108.2
C5—C4—C3	120.7 (6)	C40A—C39A—H39C	109.7
C5—C4—H4	119.6	C40A—C39A—H39D	109.7
N5—C21—H21A	108.3	C39A—C40A—H40D	109.5
N5—C21—H21B	108.3	C39A—C40A—H40E	109.5
H21A—C21—H21B	107.4	C39A—C40A—H40F	109.5
C22—C21—N5	116.1 (6)	H40D—C40A—H40E	109.5
C22—C21—H21A	108.3	H40D—C40A—H40F	109.5
C22—C21—H21B	108.3	H40E—C40A—H40F	109.5
C19—C20—H20A	109.5	N6—C33A—H33C	107.9
C19—C20—H20B	109.5	N6—C33A—H33D	107.9
C19—C20—H20C	109.5	H33C—C33A—H33D	107.2
H20A—C20—H20B	109.5	C34A—C33A—N6	117.8 (18)
H20A—C20—H20C	109.5	C34A—C33A—H33C	107.9
H20B—C20—H20C	109.5	C34A—C33A—H33D	107.9
C21—C22—H22A	109.5	C33A—C34A—H34C	110.6
C21—C22—H22B	109.5	C33A—C34A—H34D	110.6
C21—C22—C23	110.6 (7)	C33A—C34A—C35A	106 (2)
H22A—C22—H22B	108.1	H34C—C34A—H34D	108.8
C23—C22—H22A	109.5	C35A—C34A—H34C	110.6
C23—C22—H22B	109.5	C35A—C34A—H34D	110.6
N6—C45—H45A	108.3	C34A—C35A—H35C	109.4
N6—C45—H45B	108.3	C34A—C35A—H35D	109.4
H45A—C45—H45B	107.4	H35C—C35A—H35D	108.0
C46—C45—N6	115.9 (7)	C36A—C35A—C34A	111 (3)
C46—C45—H45A	108.3	C36A—C35A—H35C	109.4
C46—C45—H45B	108.3	C36A—C35A—H35D	109.4
C43—C42—H42A	109.5	C35A—C36A—H36D	109.5
C43—C42—H42B	109.5	C35A—C36A—H36E	109.5
C43—C42—C41	110.6 (12)	C35A—C36A—H36F	109.5
H42A—C42—H42B	108.1	H36D—C36A—H36E	109.5
C41—C42—H42A	109.5	H36D—C36A—H36F	109.5
C41—C42—H42B	109.5	H36E—C36A—H36F	109.5
N6—C33—H33A	108.4	N6—C41A—H41C	109.9
N6—C33—H33B	108.4	N6—C41A—H41D	109.9
H33A—C33—H33B	107.4	N6—C41A—C42A	108.9 (15)
C34—C33—N6	115.6 (6)	H41C—C41A—H41D	108.3
C34—C33—H33A	108.4	C42A—C41A—H41C	109.9
C34—C33—H33B	108.4	C42A—C41A—H41D	109.9
N5—C25—H25A	108.4	C41A—C42A—H42C	109.8
N5—C25—H25B	108.4	C41A—C42A—H42D	109.8
H25A—C25—H25B	107.5	C41A—C42A—C43A	109.4 (19)
C26—C25—N5	115.4 (8)	H42C—C42A—H42D	108.2
C26—C25—H25A	108.4	C43A—C42A—H42C	109.8
C26—C25—H25B	108.4	C43A—C42A—H42D	109.8
N6—C41—H41A	108.6	C42A—C43A—H43C	109.2
N6—C41—H41B	108.6	C42A—C43A—H43D	109.2
C42—C41—N6	114.7 (6)	C42A—C43A—C44A	112 (2)
C42—C41—H41A	108.6	H43C—C43A—H43D	107.9
C42—C41—H41B	108.6	C44A—C43A—H43C	109.2
H41A—C41—H41B	107.6	C44A—C43A—H43D	109.2
H35A—C35—H35B	108.5	C43A—C44A—H44D	109.5
C34—C35—H35A	110.3	C43A—C44A—H44E	109.5
C34—C35—H35B	110.3	C43A—C44A—H44F	109.5
C36—C35—H35A	110.3	H44D—C44A—H44E	109.5
C36—C35—H35B	110.3	H44D—C44A—H44F	109.5
C36—C35—C34	107.2 (12)	H44E—C44A—H44F	109.5
H24A—C24—H24B	109.5	N6—C45A—H45C	107.5
H24A—C24—H24C	109.5	N6—C45A—H45D	107.5
H24B—C24—H24C	109.5	N6—C45A—C46A	119.5 (19)
C23—C24—H24A	109.5	H45C—C45A—H45D	107.0
C23—C24—H24B	109.5	C46A—C45A—H45C	107.5
C23—C24—H24C	109.5	C46A—C45A—H45D	107.5
H39A—C39—H39B	108.3	C45A—C46A—H46C	109.1
C38—C39—H39A	109.9	C45A—C46A—H46D	109.1
C38—C39—H39B	109.9	C45A—C46A—C47A	113 (3)
C38—C39—C40	108.8 (11)	H46C—C46A—H46D	107.8
C40—C39—H39A	109.9	C47A—C46A—H46C	109.1
C40—C39—H39B	109.9	C47A—C46A—H46D	109.1
H47A—C47—H47B	107.2	C46A—C47A—H47C	106.9
C46—C47—H47A	108.0	C46A—C47A—H47D	106.9
C46—C47—H47B	108.0	H47C—C47A—H47D	106.7
C48—C47—H47A	108.0	C48A—C47A—C46A	122 (3)
C48—C47—H47B	108.0	C48A—C47A—H47C	106.9
C48—C47—C46	117.3 (18)	C48A—C47A—H47D	106.9
N5—C29—H29A	108.4	C47A—C48A—H48D	109.5
N5—C29—H29B	108.4	C47A—C48A—H48E	109.5
H29A—C29—H29B	107.5	C47A—C48A—H48F	109.5
C30—C29—N5	115.5 (10)	H48D—C48A—H48E	109.5
C30—C29—H29A	108.4	H48D—C48A—H48F	109.5
C30—C29—H29B	108.4	H48E—C48A—H48F	109.5
C43—C44—H44A	109.5		
			
Bi2—N2—C16—C15	166.4 (5)	C11—C6—C7—N3	178.1 (7)
Bi2—N2—C12—C13	−166.9 (6)	C17—N5—C21—C22	59.4 (8)
Bi1—N1—C1—C2	−170.9 (5)	C17—N5—C25—C26	−55.7 (11)
Bi1—N1—C5—C4	170.6 (5)	C17—N5—C29—C30	−171.5 (10)
S1—N4—C8—C7	0.8 (8)	C17—N5—C29A—C30A	180 (2)
S1—N4—C8—C9	178.9 (6)	C17—C18—C19—C20	179.8 (7)
N4—S1—N3—C7	1.6 (6)	C4—C3—C6—C7	157.6 (7)
N4—C8—C9—C10	−179.2 (7)	C4—C3—C6—C11	−23.0 (10)
N4—C8—C9—C14	1.2 (11)	C4—C3—C2—C1	−1.0 (9)
N6—C45—C46—C47	−177.1 (13)	C21—N5—C17—C18	62.9 (8)
N6—C33—C34—C35	−164.9 (9)	C21—N5—C25—C26	−176.7 (9)
N6—C37—C38—C39	179.6 (8)	C21—N5—C29—C30	−53.1 (13)
N6—C37A—C38A—C39A	−171 (2)	C21—N5—C29A—C30A	−53 (3)
N6—C33A—C34A—C35A	−148 (3)	C21—C22—C23—C24	178.4 (8)
N6—C41A—C42A—C43A	171 (4)	C45—N6—C33—C34	66.7 (9)
N6—C45A—C46A—C47A	−172 (4)	C45—N6—C41—C42	−174.9 (8)
N1—C1—C2—C3	0.1 (10)	C45—N6—C37—C38	59.4 (9)
N1—C5—C4—C3	−1.3 (11)	C33—N6—C45—C46	175.0 (8)
N5—C21—C22—C23	172.0 (7)	C33—N6—C41—C42	−57.6 (9)
N5—C25—C26—C27	179.1 (9)	C33—N6—C37—C38	−63.5 (9)
N5—C29—C30—C31	−164.6 (12)	C25—N5—C17—C18	−55.3 (10)
N5—C29A—C30A—C31A	162 (3)	C25—N5—C21—C22	−179.4 (7)
C3—C6—C7—C8	179.2 (6)	C25—N5—C29—C30	64.1 (14)
C3—C6—C7—N3	−2.5 (11)	C25—N5—C29A—C30A	65 (3)
C3—C6—C11—C10	−179.0 (6)	C41—N6—C45—C46	−67.5 (10)
C6—C3—C2—C1	179.6 (6)	C41—N6—C33—C34	−48.9 (9)
C6—C3—C4—C5	−179.0 (6)	C41—N6—C37—C38	176.7 (7)
C6—C7—C8—N4	178.8 (6)	C29—N5—C17—C18	177.7 (9)
C6—C7—C8—C9	0.7 (10)	C29—N5—C21—C22	−56.1 (9)
C6—C7—N3—S1	−179.7 (6)	C29—N5—C25—C26	66.4 (12)
C7—C6—C11—C10	0.4 (10)	C29—C30—C31—C32	74.2 (17)
C7—C8—C9—C10	−1.3 (10)	C44—C43—C42—C41	−75 (2)
C7—C8—C9—C14	179.1 (6)	C37—N6—C45—C46	50.9 (10)
C8—C7—N3—S1	−1.3 (8)	C37—N6—C33—C34	−167.9 (7)
C8—C9—C10—C11	1.5 (10)	C37—N6—C41—C42	63.4 (9)
C8—C9—C14—C13	16.8 (10)	C40—C39—C38—C37	−178.1 (12)
C8—C9—C14—C15	−164.4 (6)	C36—C35—C34—C33	−178.1 (11)
N2—C16—C15—C14	1.0 (11)	C28—C27—C26—C25	89.3 (17)
N3—S1—N4—C8	−1.4 (6)	C28A—C27—C26—C25	163.2 (18)
N3—C7—C8—N4	0.3 (9)	C48—C47—C46—C45	70 (2)
N3—C7—C8—C9	−177.8 (6)	C29A—N5—C17—C18	−161.6 (16)
C1—N1—C5—C4	0.3 (10)	C29A—N5—C21—C22	−63.9 (16)
C13—C14—C15—C16	−0.7 (10)	C29A—N5—C25—C26	51.9 (14)
C2—C3—C6—C7	−23.0 (10)	C29A—C30A—C31A—C32A	72 (4)
C2—C3—C6—C11	156.4 (7)	C37A—N6—C33A—C34A	−63 (2)
C2—C3—C4—C5	1.5 (10)	C37A—N6—C41A—C42A	163 (2)
C9—C10—C11—C6	−1.1 (12)	C37A—N6—C45A—C46A	−52 (3)
C9—C14—C15—C16	−179.6 (6)	C37A—C38A—C39A—C40A	−67 (5)
C10—C9—C14—C13	−162.8 (7)	C33A—N6—C37A—C38A	−171 (2)
C10—C9—C14—C15	16.0 (10)	C33A—N6—C41A—C42A	51 (3)
C43—C42—C41—N6	172.1 (10)	C33A—N6—C45A—C46A	56 (3)
C14—C13—C12—N2	−1.8 (13)	C33A—C34A—C35A—C36A	−179 (4)
C14—C9—C10—C11	−178.9 (6)	C41A—N6—C37A—C38A	68 (2)
C5—N1—C1—C2	0.2 (10)	C41A—N6—C33A—C34A	51 (3)
C16—N2—C12—C13	1.9 (12)	C41A—N6—C45A—C46A	−171 (3)
C12—N2—C16—C15	−1.5 (11)	C41A—C42A—C43A—C44A	177 (5)
C12—C13—C14—C9	180.0 (7)	C45A—N6—C37A—C38A	−58 (2)
C12—C13—C14—C15	1.1 (11)	C45A—N6—C33A—C34A	−175 (2)
C19—C18—C17—N5	174.1 (7)	C45A—N6—C41A—C42A	−78 (3)
C11—C6—C7—C8	−0.2 (10)	C45A—C46A—C47A—C48A	62 (8)

Symmetry codes: (i) *x*, *y*−1, *z*; (ii) *x*, *y*+1, *z*.

### catena-Poly[[triiodidobismuth(III)]-di-µ-iodido-[triiodidobismuth(III)]-µ-4,7-bis(pyridin-4-yl)benzo[c][1,2,5]thiadiazole] Hydrogen-bond geometry (Å, º)

*Cg* is the centroid of the benzothiadiazole ring.

**Table:**

*D*—H···*A*	*D*—H	H···*A*	*D*···*A*	*D*—H···*A*
C39^iii^—H39*D*^iii^···I3	0.97	2.98	3.76 (4)	157
C37^iii^—H37*A*^iii^···I3	0.99	3.03	3.966 (8)	138
C34^iii^—H34*D*^iii^···I4	0.99	2.89	3.71 (3)	141
C36^iii^—H36*E*^iii^···I4	0.98	2.48	3.41 (5)	158
C37^i^—H37*C*^i^···I7	0.99	3.03	3.96 (2)	157
C40—H40*A*···*Cg*	0.98	2.86	3.56 (2)	129

Symmetry codes: (i) *x*, *y*−1, *z*; (iii) −*x*+1, *y*+1/2, −*z*+1/2.
